# Study of Sperm Reproductive Parameters in Mature Zanjani Viper

**Published:** 2014-05-25

**Authors:** Malihe Moshiri, Fatemeh Todehdehghan, Abdolhossein Shiravi

**Affiliations:** 1 Department of Basic Science, Islamic Azad University, Damghan Branch, Damghan, Iran; 2 Department of Venomous Animals and Antivenin Production, Vaccine and Serum Research Institute, Karaj, Iran

**Keywords:** Sperm, Basic Reproductive Rate, Snake, Viper, Iran

## Abstract

**Objective:**

Zanjani viper (*Vipera albicornuta*) is an endemic venomous snake in East Azerbai- jan Province, Iran which is medically important due to its application for antivenin production in the laboratory. We need to produce this snake in captivity. This study was conducted to charac- terize mature male Zanjani viper and to evaluate its sperm reproductive parameters.

**Materials and Methods:**

This applied- descriptive study was conducted on twenty Zan- jani viper samples collected from Ag Dag Mountain in East Azarbaijan Province, Iran, between September and October 2010. After the snakes were anesthetized and sacrificed humanly, their morphometric specifications and sperm reproductive parameters, including concentration, motility, vitality, morphology, and survival time, were measured.

**Results:**

Morphometric specifications and evaluation of sperms of the snake showed the following information: Zanjani male viper, body length of 73.65 ± 4.35 cm, tail length of 5.465 ± 0.48 cm, and mature snakes with testicular volumes of 0.61 ± 0.81 ml (right) and of 0.46 ± 0.17 ml (left). Our findings revealed average sperm concen- tration of 0.47 ± 0.1 ×10^6^ml^-1^, motility of 49 -55 %, vitality of 46.11 ± 9.63 %, normal morphology of 61.71 ± 5.3%, and survival time of 6 ± 2 hours at the laboratory tem- perature. Statistical analyses were performed using Student’s t test for comparison of two values, and one-way ANOVA was applied where three values were compared.

**Conclusion:**

Results suggest that mature Zanjani male viper with mature sperms in its vas deferens is present in late summer and early autumn seasons in Bostanabad County, Iran.

## Introduction

Reproductive-physiological studies on animal species provide solutions to wildlife conservation and also contribute with useful data for research on economically important animals (1). Particularly, evaluation of sperm quality provides a powerful tool for the gene banks creation and species conservation in captivity and nature; furthermore, it determines the male fertility potential (2) providing remarkable contributions for areas of assisted reproduction (3). *Vipera albicornuta*, as an endemic venomous snake to Iran, is founded in different parts of Alborz Mountains, Northern Zagros Mountains, Gillian Province, East Azerbaijan Province, Ghazvin County, and specially Zanjan County (4, 5). No subspecies has been yet reported for *Vipera albicornuta*. Zanjani viper is hunted from wild and carried to the laboratory for production of biomedical products, especially for venom and antivenin production. In order to avoid environmental modification, researchers recommend population increment in captivity (6, 7). To do so, we need to study snake’s reproductive biology. This research was carried on evaluation of sperms reproductive parameters in order to find mature male snake’s characteristics that contributes to our further study on the snake production in captivity.

## Materials and Methods

Twenty Zanjani male vipers were collected from Ag Dag Mountain located in Bostanabad County toward City of Maraghe in East Azerbaijan Province, Iran. The annual temperature of this area is between 35˚C (maximum) and -20˚C (minimum) with average raining of 300 mm/year. This applied- descriptive study was part of a project and approved by the Ethics Committee of Razi Vaccine Research Institute, and all procedures were carried out according to animal ethics guideline (8). The sperm samples were collected in September to October 2010. It is noted that in viperidae, vasa deferentia contains sperm from February to October (9). The snakes were kept in cages with 1.5 meter length, 40-60 cm width, and 70 cm height in temperature of 20-30˚C and in a 10/14 light/ dark cycle, while they were fed every second week. Snake was anesthetized with 1% lidocaine (15mgkg^-1^, Daroupakhsh, Iran) and sacrificed. The body length of snakes was measured from tip snout to vent by a measuring tape (10). The testes and vas deferens were taken out. Gonads were weighted by scale (OSK, Fx- 300, Japan), their dimensions were measured by caliper (domain of 0-150 mm) and their volumes were calculated by Cha formula (11). In order to collect sperms, the vas deferens was divided into three parts: i. initial, ii. middle, and iii. distal (12), while each part was kept in vials containing one ml phosphate buffered saline (PBS) (13) for 45 minutes. Then, sperm motility, morphology, vitality, and concentration were studied. Motility of the spermatozoa in the extended semen was estimated by placing a drop of diluted semen on a slide under a coverslip at ambient temperature (26-27˚C) and by estimating the percentage of progressively motile sperm cells to the nearest 5% in five different microscopic fields under ×400 magnification. In order to study the sperm motility, the following four grades were considered: i. A=quick progressive in straight paths, ii. B=slow progressive in straight or not straight paths, iii. C=motile in place and iv. D=immotile (7, 14). To determine the concentration, a portion of the extended sperm sample was further diluted to 1:10 in PBS, and the sperm cells were counted in both chambers of a hemacytometer under a phase contrast microscopy ×400 (Olympus, Japan) (15). Concentration of the sperm was calculated according to a method presented by Rashidi et al. (16). Morphology was evaluated by observing 100 sperm cells under a microscope (Olympus, Japan) (×1000) (17, 18). Zanjani viper sperms like sperms of other vipers are filiform with narrow curved head (13, 19- 21), spicule-shaped tips in the acrosomes (19), short mid-piece (with a compact electron-dense nucleus), and long tail (very slender) (18, 21), which all sperms with such morphology were considered normal. The survival time of sperm cells was measured at the laboratory temperature of 23 ± 2˚C (13, 15, 18).

### Statistical analysis

Student’s t test was performed for comparison of two values, and one-way ANOVA was applied where three values were compared.

## Results

Morphological specifications of Zanjani viper are shown in table 1. Testes are oval-shape, elongated with light pink color, surrounded with many vessels on the surface, and laid in the one third of posterior part of body. In addition, right testis is located upper than the left one and is bigger than left one, so their weight and length (Table 1) are significantly different at p≤0.001. Average volume of right testis also was greater than left testis at p<0.001. Length of left vas deferens was larger than right one at p<0.001. Sperms were present in three regions of vas deferens. Sperm reproductive parameters are shown in tables 2 and 3, while statistical comparisons are assigned by asterisks.

**Table 1:** Morphometric specifications of Zanjani male viper (Mean ± SD)


Variable	Value

**Body weight (g)**	193.25 ± 39.54
**Body length , from snout to vent (cm)**	73.65 ± 4.35
**Tail length (cm)**	5.465 ± 0.48
Length of right vas deferens (cm)***	20.85 ± 1.71
Length of left vas deferens (cm)***	18.03 ± 2.05
Weight of right testis (g) ***	0.65 ± 0.189
Weight of left testis (g) ***	0.358 ± 0.16
Volume of right testis (ml) ***	0.61 ± 0.81
Volume of left testis (ml) ***	0.46 ± 0.17


***; At p≤0.001, differences are considered to be statistically significant

**Table 2:** Sperms concentration, vitality, survival and morphology in Zanjani viper, (Mean ± SD)


Variable	Value

Sperm concentration in the first region of right duct(×10^6^ ml^-1^)*	0.27 ± 0.2
Sperm concentration in the middle region of right duct(×10^6^ ml^-1^)*	0.47 ± 0.1
Sperm concentration in the final region of right duct(×10^6^ ml^-1^)*	0.41 ± 0.2
Sperm concentration in the first region of left duct(×10^6^ ml^-1^)*	0.16 ± 0.01
Sperm concentration in the middle region of left duct(×10^6^ ml^-1^)*	0.21 ± 0.1
Sperm concentration in the final region of left duct(×10^6^ ml^-1^)*	0.30 ± 0.1
Live sperm (first region of duct) (%)*	44.16 ± 16.65
Live sperm (middle region of duct) (%)*	44.44 ± 12.7
Live sperm (final region of duct) (%)*	46.11 ± 9.63
Dead sperm (first region of duct) (%)*	44.72 ± 16.84
Dead sperm (middle region of duct) (%)*	50 ± 13.93
Dead sperm (final region of duct) (%)*	53.88 ± 9.63
**Sperm survival time (hours)**	6.00 ± 2.00
Normal sperm (%) ***	61.71± 5.3
Abnormal sperm (%) ***	38.28 ± 5.3
**Abnormal head less (%)**	2 ± 0.7
**Abnormal folded tail (%)**	9.11 ± 3.0
**Abnormal bent head (%)**	5.16 ± 1.5
**Abnormal swollen head (%)**	5.16 ± 0.7
**Abnormal spiral tail (%)**	4.94 ± 1.6
**Abnormal ring tail (%)**	11.91±2.4


*; At p>0.05, there is no significant differences and ***; Significantly different at p<0.0001.

**Table 3:** Sperm motility rate in three different parts of vas deferns duct in Zanjani viper


Motile sperm in first region of right duct (%)**	50.62(0-63)	**Left duct (%)**	51.14(48-54)
**Grade A**	2.06(0-13)		1.42(0-7)
**Grade B**	1.93(0-4)		2.143(0-4)
**Grade C**	46.62(0-54)		47.57(43-51)
**Grade D (immotile sperm) (%)**	49.37(0-75)		48.86(46-52)
Motile sperm in middle region of right duct (%)**	54(0-66)	**Left duct (%)**	50(48-52)
**Grade A**	4.58(0-13)		0.38(0-3)
**Grade B**	3.29(0-8)		2.00(0-3)
**Grade C**	46.11(0-53)		47.63(44-49)
**Grade D (immotile sperm) (%)**	46(0-57)		50(48-52)
Motile sperm in final region of right duct (%)**	55(0-85)	**Left duct (%)**	49.37(44-54)
**Grade A**	4.16(0-27)		0.00
**Grade B**	3.83(0-13)		2.75(0-8)
**Grade C**	47(37-57)		46.62(42-54)
**Grade D (immotile sperm) (%)**	45 (15-53)		50.62(46-56)


**There is no significant difference in motility rate among three different parts of ducts at p> 0.05.

## Discussion

In male reptiles, storage of sperm is in the vas deferens, and it is different from mammals (22, 23). Sperm storage is a common characteristic of family Viperidae (24, 25). Previous studies have indicated that the number of motile spermatozoa and their movement speed is directly correlated with fertilization success (24-26). Numbers and motility of the sperms along the vas deferens in the Crotalus durissus terrificus from southeastern Brazil were seasonally changed (19, 27). Although spermatozoa are present in C. durissus terrificus all the year, sperms counts are the lowest during winter and the highest in summer and autumn, (28, 29) which is similar to sperms counts in Zanjani viper in late summer and early autumn seasons. This may be due to the high temperatures favoring testicular recrudescence in early spring when spermatogenesis begins (29). Our data demonstrate that the storage pattern in the vas deferens is opposite to tropical nonhibernator C. durissus terrificus, hibernator north American species C. viridis, C. scutelatus, C. atrox, and A. piscivorus (24, 25). Contrary to our finding, there are no differences in the sperms motility rate throughout the vas deferens. Different studies on C. durissus terrificus have showed that motility of sperm increase from the proximal to the distal region of the vas deferens (15) and reaches to the maximum of their motility in the distal region. Yokoyama and Yoshida (27) observed sperm storage in posterior region of the vas deferens in the Japanese Trimeresurus flavoviridis. Spermatozoa of B. c. occidentalis share a similar morphology with the general model described for snakes by Oliver et al. (18). These filiform cells contain a narrow curved head with its anterior portion covered by a small conic acrosome (20) that is similar to our finding. It has been reported that sperm morphologic abnormalities have the greatest negative correlation to fertility in farm animals, and heat stress is the main cause of sperm abnormalities in these animals. The effect of heat stress on reptile’s spermatozoa has not been evaluated. Since reptiles are ectothermic animals, they depend on their environmental temperature to regulate their core temperature and sperm parameters (30). It would not be unexpected for a Zanjani snake to experience a 2.7-5.5˚C (10-15˚F) difference in body temperature in a given day; therefore, semen production would decrease and sperm abnormalities would increase in snakes experiencing a large drop in body temperature, although it has not been evaluated, Semen collected manually from an Angolan python (*Python anchietae*) and Timor python (*Python timoriensis*) had a volume of 0.1-0.4 ml and a concentration of approximately 1,500×10^6^ sperm cells/ml (7). Semen collected manually from the Brazilian rattlesnake had a volume, motility, and concentration of 0.015 ml, 70%, and 1,522×10^6^ sperm cells/ml, respectively (28). Checkered garter snake semen collected by electroejaculation was found to have a volume range of 0.05-0.10 ml and motility of 50-70%. In corn snake (Elaphe Guttata), sperm motility was 92.5% and related concentration was 825×10^6^ sperm cells/ml (17). In our study, the motility of sperm produced by Zanjani snakes was almost similar to the garter snakes and rattlesnakes, but lower than corn snake. The lower concentration of sperm in the Zanjani snakes could be attributed to the reproductive behavior of these snakes. To date, no studies have been published on the collection and characterization of spermatozoa from mature Zanjani vipers (*Vipera albicornuta*). However, further study is in the process.

## Conclusion

The results of this work helps in the optimization of protocols for sperm collection in snake and in the establishment of basic values which would be useful to evaluate the reproductive potential of the mature Zanjani male viper that contributes to snake’s captive production.

## References

[1] Busso JM JM, Ponzio MF, Chiaraviqlio M, Fiol de Cuneo M, Ruiz RD (2005). Electroejaculation in the Chinchilla (Chinchilla
lanigera): effects of anesthesia on seminal characteristics. Res Vet Sci.

[2] Goeritz F, Quest M, Wagener A, Fassbender M, Broich A, Hildebrandt TB (2003). Seasonal timing of sperm production in roe deer: interrelationship among changes in ejaculate parameters, morphology and function of testis and accessory glands. Theriogenology.

[3] Howard JG, Bush M, Morton C, Morton F, Wentzel K, Wildt DE (1991). Comparative semen cryopreservation in ferrets (Mustela putorius furo) and pregnancies after laparoscopic intrauterine insemination with frozen-thawed spermatozoa. J Reprod Fertil.

[4] Latifi M (2000). Iran’s snakes.

[5] Latifi M (1991). The snakes of Iran.

[6] Fahrig BM, Mitchell MA, Eilts BE, Paccamonti DL (2007). Characterization and cooled storage of semen from Corn snakes (Elaphe guttata). J Zoo Wildl Med.

[7] Mengden AG, Platz CG, Hubbard R, Quinn H, Murphy JB, Collins JT (1980). Semen collection, freezing, and artificial insemination in snakes. Contributions to herpetology reproductive biology and diseases of captive reptiles.

[8] Institute of Standards and Industrial Research of Iran (2008). Biological evaluation of medical devices-Part 2: Animal welfare Requirements. ISIRI.

[9] Goldberg SR (2007). Testicular cycle of the Western Diamondback Rattlesnake, Crotalus atrox (Serpentes: Viperidae), from Arizona. Bull Md Herpetol Soc.

[10] Blouin-Demer G (2003). Precision and accuracy of body-size measurements in a constricting, large-body snake (Elaph obsolete). Herpetol Rev.

[11] Cha MH, Ahn BC, Kim YS (2006). Inaccuracy in ultrasonographic measurement of the testicular volume in children. Korean J Urol.

[12] Sever DM (2010). Ultrastructure of the reproductive system of the Black Swamp Snake (Seminatrix pygaea).IV.Occurrence of an ampulla ductus deferentis. J Morphol.

[13] Tourmente M, Cardozo GA, Guidobaldi HA, Giojalas LC, Bertona M, Chiaraviglio M (2007). Sperm motility parameters to evaluate the seminal quality of Boa constrictor oceidentalis, a threatened snake species. Res Vet Sci.

[14] Dada R, Gupta NP, Kucheria K (2001). Deteriration of sperm morphology in men exposed to high temperature. J Anat Soc India.

[15] Almeida- Santos SM, Abdalla FMF, Silveira PF, Yamanouye N, Berno MC, Salomao MG (2004). Reproductive cycle of the neotropical crotalus durissus terrificus: I.seasonal levels and interplay between steroid hormones and vasotocinas. Gen Comp Evdocer.

[16] Rashidi I, Movahedin M, Tiraihi T (2004). The effects of pentoxifylline on mouse epididymal sperm parameters, fertilization and cleavage rate after short time preservation. Iran J Reprod Med.

[17] Fahrig BM, Mitchell MA, Eilts BE, Paccamonti DL (2007). Characterization and cooled storage of semen from Corn snakes (Elaphe guttata). J Zoo Wildl Med.

[18] Oliver S, Jamieson BGM, Scheltinga D (1996). The ultrastructure of spermatozoa of Squamata.II.Agamidae, Varanidae, Colubridae, Elapidae and Boidae (Reptilia). Herpetologica.

[19] Almeida-Santosa SM, Iara L, Marta LF, Antoniazzib M, Jared C (2004). Sperm storage in males of the snake Crotalus durissue terrificus (Crotalinae: Viperidae) in sortheastern Brazil. Comp Biochem Phys A.

[20] Tourmente M, Cardozo G, Bertona M, Guidobaldi A, Giojalas L, Chiaraviglio M (2006). The ultrastructure of the spermatozoa of Boa constrictor occidentalis, with considerations on its mating system and sperm competition theories. Acta Zool.

[21] Tourmente M, Gomendio M, Roldan ERS, Giojalas LC, Chiaraviglio M (2009). Sperm competition and reproductive mode influence sperm dimensions and structure among anakes. Evolution.

[22] Jones RC (1998). Evolution of the vertebrate epididymis. J Reprod Fertil Suppl.

[23] Sever DM, Stevens RA, Ryan TJ, Hamlett WC (2002). Ultrastructure of the reproductive system of the black swamp snake (Seminatrix pygaea): III.Sexual segment of the male kidney. J Morphol.

[24] Aldridge RD, Duvall D (2002). The evolution of the mating season in the pitvipers of North America. Herpetol Monogr.

[25] Schuett GW, Campbell JA, Brodie ED Jr (1992). Is long-term sperm storage an important component of the reproductive biology of temperate pitvipers?. Biology of the Pitvipers.

[26] Gist D, Turner T, Congdon J (2000). Chemical and thermal effects on the viability and motility of spermatozoa from the turtle epididimys. J Reprod Fertil.

[27] Yokoyama F, Yoshida H (1993). The reproductive cycle of the male Habu, Trimeresurus flavoviridis. The Snake.

[28] Zacariotti RL, Grego KF, Fernandes W, Santanna SS, de Barros Vaz Guimarães MA (2007). Semen collection and evaluation in free-ranging Brazilian rattlesnakes (Crotalus durissus terrificus). Zoo Biol.

[29] Almeida-Santos SM, Orsi AM (2002). Reproductive cycle in Crotalus durissus and Bothrops jararaca (Serpentes, Viperidae): morphology and oviductal function. Rev Bras Reprod Anim.

[30] Tourmente M, Giojalas LC, Chiaraviqlio M (2011). Sperm parameters associated with reproductive ecology in two snake species. Herpetologica.

