# Study of Sperm Reproductive Parameters in Mature Zanjani Viper

**Published:** 2014-10-04

**Authors:** Malihe Moshiri, Fatemeh Todehdehghan, Abdolhossein Shiravi

**Affiliations:** 1 Department of Basic Science, Islamic Azad University, Damghan Branch, Damghan, Iran; 2 Department of Venomous Animals and Antivenin Production, Vaccine and Serum Research Institute, Karaj, Iran

In this article which was published in Cell J, Vol 16, No 2, Summer 2014, on pages 111-116, the title of table 3 was published incorrectly as: "Sperms concentration, vitality, survival and morphology in Zanjani viper, (mean ± SD)". The correct one is "Sperm motility rate in three different parts of vas deferns duct in Zanjani viper".

**Table 3:** Sperm motility rate in three different parts of vas deferns duct in Zanjani viper


Motile sperm in first region of right duct (%)**	50.62(0-63)	**Left duct (%)**	51.14(48-54)
**Grade A**	2.06(0-13)		1.42(0-7)
**Grade B**	1.93(0-4)		2.143(0-4)
**Grade C**	46.62(0-54)		47.57(43-51)
**Grade D (immotile sperm) (%)**	49.37(0-75)		48.86(46-52)
Motile sperm in middle region of right duct (%)**	54(0-66)	**Left duct (%)**	50(48-52)
**Grade A**	4.58(0-13)		0.38(0-3)
**Grade B**	3.29(0-8)		2.00(0-3)
**Grade C**	46.11(0-53)		47.63(44-49)
**Grade D (immotile sperm) (%)**	46(0-57)		50(48-52)
Motile sperm in final region of right duct (%)**	55(0-85)	**Left duct (%)**	49.37(44-54)
**Grade A**	4.16(0-27)		0.00
**Grade B**	3.83(0-13)		2.75(0-8)
**Grade C**	47(37-57)		46.62(42-54)
**Grade D (immotile sperm) (%)**	45 (15-53)		50.62(46-56)


**There is no significant difference in motility rate among three different parts of ducts at p>0.05.

